# Announcing the new Editor-in-Chief of *e*cancermedicalscience

**DOI:** 10.3332/ecancer.2024.ed133

**Published:** 2024-06-10

**Authors:** Eduardo Cazap, Enrique Soto Pérez de Celis

**Affiliations:** 1 ecancer, 13 King Square Avenue, Bristol, BS2 8HU, UK; 2 Slacom, Cordoba 2415, Buenos Aires, Argentina; 3 Division of Medical Oncology, University of Colorado Anschutz Medical Campus, Aurora, CO, USA; 4 Department of Geriatrics, Instituto Nacional de Ciencias Médicas y Nutrición Salvador Zubirán, Mexico City, Mexico

## Welcome to Enrique Soto Pérez de Celis, new Editor-in-Chief

Five years ago, I started my term as Editor-in-Chief of ecancermedicalscience, building on the foundations laid down by Professors Umberto Veronesi and Gordon McVie. Why did I decide to accept such a challenging proposal?

At that time, I noticed a high proportion of high-level, English-language, costly publications in the landscape of oncology journals. However, I wondered what was happening to the urgently needed science, experience, data, and information from developing countries and regions of the world. This lack of information makes it extremely difficult to make both clinical decisions and healthcare or public policy planning. The simple extrapolation of data from high-income countries (HICs) without the corresponding information regarding local clinical, genetic, or molecular characteristics, availability of healthcare resources, and cultural and/or social barriers is insufficient to make appropriate therapeutic choices. My objective was to build ecancermedicalscience as a leading journal for researchers from developing countries, reducing global inequalities in cancer care and control, and providing free access to all articles from the moment of publication. ecancermedicalscience would only charge authors who had access to dedicated funding to cover publication costs 1.

In 2020 the Board of the ecancer Foundation decided that ecancermedicalscience would only accept submissions featuring at least one author from a low- and middle-income country (LMIC), or those having a significant impact on underserved settings 2. This has been instrumental in effectively pursuing our objective of facilitating and promoting the submission of data from LMICs, many of which have never been previously reported in the world literature. Another critical component that has moved forward our mission is the fact that ecancermedicalscience accepts manuscripts in both English and Spanish. Additionally, Spanish-to-English translation of accepted articles is provided with no charge to the authors 1. As a result of these policies, in the last five years over 600 authors from more than 30 LMICs have published manuscripts in ecancermedicalscience.

The impact of the journal in its 17 years of existence has been considerable. ecancermedicalscience’s manuscripts have over 50,000 PubMed Central downloads each month, there are 21,000 registered members, and our editorial board is made up of 138 distinguished scientists and clinicians from around the world. In addition, *e*cancer has the largest library of key opinion leader oncology video interviews in the world, which in the last 12 years has received over 20 million visits.

To ensure the proper evaluation and review of submitted articles, *e*cancermedicalscience’s Editorial Board and reviewers comply with a policy that recognises the characteristics, environment, and culture of cancer care and global health systems. *e*cancermedicalscience believes that good information and research originating in developing countries can be greatly beneficial for patients and health systems in HICs in terms of cost savings, access to care, and reduction of inequalities for immigrants and other underserved populations. We certainly live in demanding times: new globalised channels of information appear every day; population changes and migrations impact the epidemiology of cancer; and the exponential growth of technology has not benefited all populations in the same way.

Looking to the future, *e*cancermedicalscience needs an Editor-in-chief who is responsive to the challenges represented by the myriad of updates, changes, and innovations necessary to maintain and improve *e*cancermedicalscience’s leading role in the international context. To that end, and after a careful and profound analysis of possible candidates, the name of Dr Enrique Soto Pérez de Celis came to the table. Enrique is a geriatric oncologist and Associate Professor of Medical Oncology at the University of Colorado Anschutz, and the Associate Director of Global Oncology for the University of Colorado Cancer Center. He previously worked in the Department of Geriatrics at the Instituto Nacional de Ciencias Médicas y Nutrición Salvador Zubirán in Mexico City, where he developed the first geriatric oncology clinic in Spanish-speaking Latin America.

Enrique’s achievements, expertise, and contributions have significantly impacted the landscape of medical oncology. These credentials have led him to hold the prominent position of member of the ASCO Board of Directors, where he has stood out for his numerous contributions in different areas of the organisation, particularly global oncology. Apart from his academic pursuits, Dr Soto Pérez de Celis is deeply committed to mentorship and education, fostering the growth of future generations. His passion for teaching and mentorship shines through in his interactions with students and colleagues alike, inspiring others to strive for excellence in their pursuits. However, one of Enrique’s most notable abilities is his prolific performance as the author of more than 160 scientific papers in top-level journals, along with his recognised and prolific performance as a reviewer, associate editor, and editor in various publications.

Due to all these previous considerations and others of similar value, the Board of Directors of the *e*cancer Foundation has named Enrique Soto Pérez de Celis as the new Editor-in-Chief of *e*cancermedicalscience. I am sure that Enrique will succeed in bringing along further improvements in the academic prestige of *e*cancermedicalscience. As a long-time contributor to the journal, I will continue collaborating with *e*cancer and *e*cancermedicalscience as needed, and I look forward to the future under the leadership of Dr Soto Pérez de Celis.

***—Eduardo Cazap***

## Building on our successes and tackling the challenges of the future, my vision as the new Editor-in-Chief

It is an enormous honor for me to accept the position as Editor-in-Chief of *e*cancermedicalscience, and to stand on the shoulders of a giant in the field of global oncology such as Dr Eduardo Cazap. As Editor-in-Chief of *e*cancermedicalscience, Dr Cazap has grown the journal into one of the leading publications in global oncology, and into the go-to forum for researchers from LMICs around the globe. As one of those researchers, I truly understand the challenges of designing, conducting, reporting, and publishing original research in resource-constrained settings, and I strongly believe that the mission of *e*cancermedicalscience, and *e*cancer as a whole, is now more important than ever.

The exponential growth of research and innovation in cancer, coupled with the development of new technologies, has led to what can only be described as an avalanche of information. However, just as when Dr Cazap took over the journal five years ago, most of this information comes from resource-rich settings in HICs, and therefore their results may not represent the everyday clinical practices of cancer care providers working in developing countries, or even in resource-limited areas of high-income countries. In this setting, understanding the panorama of cancer care from every corner of the world, and learning about solutions and innovations implemented in countries and regions that are often overlooked by oncology publications, is of the utmost importance for healthcare workers, caregivers, and patients.

There is a growing interest in expanding cancer research to underserved populations, with recent developments showing promising outcomes. Both the European Society of Medical Oncology (ESMO) and the American Society of Clinical Oncology (ASCO) have made concerted efforts to increase the participation of members from LMICs, by reducing or waiving membership fees, by providing online access to meetings, and by creating guidelines and programmes aimed at resource-limited settings [3, 4]. ASCO has further invested in global oncology by creating and growing the Journal of Global Oncology, and by convening task forces and working groups to plan and design educational initiatives and curricula for training in global cancer control [4, 5].

Despite these advances, as we move forward, we need to be cautious about the meaning of global oncology, and about its role in the cancer world. We must not, under any circumstances, allow for the siloing of global oncology. After all, global oncology IS oncology, and cancer IS cancer, regardless of the region of the world in which it is studied. Advances in cancer care taking place in Asia, Africa, and Latin America can be as impactful for patients living in Western Europe, Australia, or the United States as the reverse. As part of my role as Editor-in-Chief, one of my priorities will be to demonstrate that research designed and conducted in LMICs can improve the care of patients across the world through reverse innovation, or the translation of research findings from lower- to higher-resourced settings. I truly believe that researchers from the developing world have the unique opportunity not only to study diseases that are less prevalent in HICs but also to improve our understanding of the influence of social and cultural determinants of health on the provision of care across the cancer continuum.

The current academic landscape in oncology is challenging for researchers in LMICs, particularly those who are starting their careers, and who face an uphill battle when trying to “compete” with researchers from HICs. Personnel shortages (research assistants, statisticians, administrators), the need to be proficient in English to publish, low salaries, lack of protected research time, a predominance of pharmaceutical- company-founded research, exorbitant open-access fees charged by many journals, and the intrinsic bias of reviewers, editors, and readers against research conducted in LMICs, are some of the many hurdles LMIC-based researchers must overcome to conduct and disseminate their research.

Changes to this unequal cancer research ecosystem will not happen on their own, and as a global cancer community, we must come up with solutions for every challenge. As Editor-in-Chief of *e*cancermedicalscience, and with the support of the incredibly robust educational structure of *e*cancer, I intend to make sure that we do whatever we can to tackle these barriers and help researchers and clinicians in LMICs disseminate research findings that can translate into significant improvements in the lives of patients and caregivers across the world.

To improve the planning, design, and reporting of research, I intend to spearhead a global initiative to provide biostatistical support for researchers who do not have access to a biostatistician. This is a pressing global need that I have lived firsthand both as a young researcher conducting research in Mexico, as a reviewer of grants and manuscripts written by other researchers from LMICs and as a journal editor: the lack of strong statistical foundations can kill even the best of ideas. Therefore, the goal of this volunteer-led global cancer biostats core will be to assist researchers from LMICs to bring their ideas to a publication that has the highest scientific rigor in its design and reporting.

*e*cancermedicalscience is already helping researchers publish their manuscripts in other languages, including providing free English-language translation. I intend to increase this support by using novel technologies, including artificial intelligence (AI) and large-language models. I strongly believe that AI can help close the gap between researchers in LMICs and their HIC counterparts, many of whom have easy access to librarians, proofreaders, and medical writers. By utilising AI in a responsible, ethical, and efficient manner, we can expedite and improve the reporting of research from areas of the world where these personnel are lacking. Likewise, the potential of AI for translating findings into various languages in a fast and cost-effective manner provides endless possibilities for enhancing the communication of research. As Editor-in-Chief, I will work with our staff, editorial board, and reviewers to make sure that we harness the power of AI in a way that leads to better research and better care for patients around the world.

Although improving the salaries and access to protected time of researchers in LMICs goes beyond what a journal can do, I am committed to continuing *e*cancermedicalscience’s policy of waiving all fees for authors who do not have dedicated funding for publishing in an open-access journal. This needs to happen to foster the reporting of investigator-initiated research looking at real-life outcomes that matter to patients, and not only to the reporting of pharma-led studies. At the same time, I will continue the journal’s policy of ensuring that authors from LMICs are correctly acknowledged and represented since this is one of the only ways to stop parasitic research practices. *e*cancermedicalscience’s editorial policies will continue to strongly oppose bias against LMIC researchers, and we will disseminate these policies through the various *e*cancer channels for the global oncology community to follow.

I sincerely thank Dr Cazap, Danny Burke, CEO of *e*cancer, and the wonderful editorial staff at *e*cancermedicalscience for the very warm welcome they have given me as Editor-in-Chief of the journal. I am excited about the upcoming opportunity to enhance cancer control and healthcare equity globally by improving research design, reporting, and dissemination through *e*cancer and *e*cancermedicalscience. I look forward to collaborating with the global oncology community to advance this agenda.

***—Enrique Soto Pérez de Celis***
